# Direct Measurement of Sedimentation Coefficient Distributions in Multimodal Nanoparticle Mixtures

**DOI:** 10.3390/nano11041027

**Published:** 2021-04-17

**Authors:** Claudia Simone Plüisch, Rouven Stuckert, Alexander Wittemann

**Affiliations:** Colloid Chemistry, Department of Chemistry, University of Konstanz, Universitaetsstrasse 10, D-78464 Konstanz, Germany; simone.plueisch@uni-konstanz.de (C.S.P.); rouven.stuckert@uni-konstanz.de (R.S.)

**Keywords:** nanoparticles, colloidal clusters, colloidal molecules, sedimentation, separation, classification of nanoparticles, analytical centrifugation, differential centrifugal sedimentation, disk centrifuge, density gradient centrifugation

## Abstract

Differential centrifugal sedimentation (DCS) is based on physical separation of nanoparticles in a centrifugal field prior to their analysis. It is suitable for resolving particle populations, which only slightly differ in size or density. Agglomeration presents a common problem in many natural and engineered processes. Reliable data on the agglomeration state are also crucial for hazard and risk assessment of nanomaterials and for grouping and read-across of nanoforms. Agglomeration results in polydisperse mixtures of nanoparticle clusters with multimodal distributions in size, density, and shape. These key parameters affect the sedimentation coefficient, which is the actual physical quantity measured in DCS, although the method is better known for particle sizing. The conversion into a particle size distribution is, however, based on the assumption of spherical shapes. The latter disregards the influence of the actual shape on the sedimentation rate. Sizes obtained in this way refer to equivalent diameters of spheres that sediment at the same velocity. This problem can be circumvented by focusing on the sedimentation coefficient distribution of complex nanoparticle mixtures. Knowledge of the latter is essential to implement and optimize preparative centrifugal routines, enabling precise and efficient sorting of complex nanoparticle mixtures. The determination of sedimentation coefficient distributions by DCS is demonstrated based on supracolloidal assemblies, which are often referred to as “colloidal molecules”. The DCS results are compared with sedimentation coefficients obtained from hydrodynamic bead-shell modeling. Furthermore, the practical implementation of the analytical findings into preparative centrifugal separations is explored.

## 1. Introduction

Pursuant to the International Union of Pure and Applied Chemistry, colloidal particles are defined as objects that have “a dimension roughly between 1 nm and 1 μm” [1]. This includes the nanoscale, which is associated to the size range of approximately 1 nm to 100 nm, in line with ISO/TS 80004-2:2015 specifications [2]. Colloidal particles underlie Brownian motion. The latter prevails over sedimentation if the nanoparticles do not form agglomerates with dimensions beyond the colloidal domain. However, agglomeration and heteroaggregation of colloidal particles are major and common problems in many natural and engineered processes [3]. Surface energy will promote the agglomeration of small particles in the absence of kinetic stabilization. The latter is strongly influenced by external parameters, such as pH, salinity, or the presence of depletants [4]. Furthermore, heteroaggregation is observed when different types of colloidal particles attract each other [5]. This can be mediated by opposite surface charges, but heteroaggregation may also occur between charged and neutral particles [6]. Attachment of macromolecules to their surface may result in bridging flocculation of colloidal particles [4,7]. In cases like these, clusters of a limited number of constituent particles are formed at the onset of nanoparticle aggregation. At this early stage of aggregation, the suspension contains a multimodal mixture of assemblies with dimensions still within the colloidal regime. An accurate analytical tool to identify the onset of aggregation has thus to provide a high resolving power for mixtures of different colloidal species.

Common particle sizing techniques such as scattering techniques permit only the precise measurement of average particle sizes of monomodal colloids or bimodal mixtures that differ by at least an order of magnitude in particle size [8]. For example, static and dynamic light scattering are inherently sensitive to larger species present in a colloidal suspension. Light scattering is thus an excellent tool to monitor the onset of aggregation because the scattered light intensity is widely governed by the aggregates, while the precursor particles only contribute slightly [9]. Other techniques, such as electron microscopy, have their strength in mapping individual aggregates [10]. However, they provide poor statistics, demand specific preparation requirements, and have harsh experimental conditions. In particular, they do not permit in-situ studies.

Analytical centrifugal methods pave the way to direct studies of water-borne colloidal particles in aqueous suspension [11]. Moreover, centrifugal separation of colloidal particles provides the extraordinary resolving power required for an in-depth analysis of polydisperse nanoparticle mixtures. Two common sedimentation techniques have been established: analytical ultracentrifugation (AUC) and differential centrifugal sedimentation (DCS). AUC is a valuable tool for analyzing structural aspects of synthetic and biological nanoparticles. It is based on integral sedimentation of colloidal particles, which means that, during the analysis, the sum (the integral) of all particles smaller than a certain size is being measured [12]. Mathematical differentiation with respect to diameter will yield the (differential) particle size distribution.

**Figure 1:**
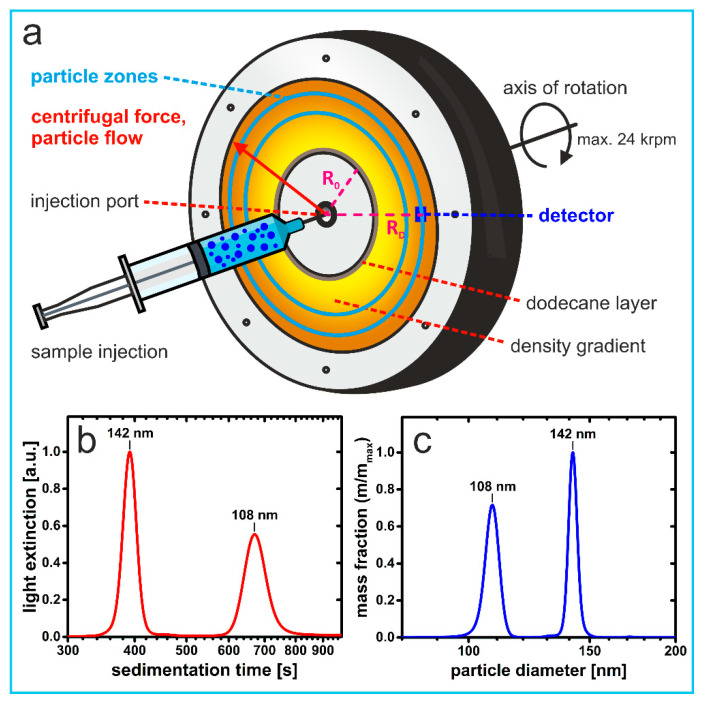
Differential centrifugal sedimentation (DCS) using a disk centrifuge: (**a**) A density gradient capped with dodecane is built within a rotating hollow, optically transparent disk. The sample is placed on top of the gradient via a central injection port. Different particle populations migrate as discrete zones at rates specified by their respective sedimentation coefficients. Attenuation of laser light is recorded continuously to quantify the scattering by particles arriving at a fixed detector position as a function of the sedimentation time. (**b**) Raw data of a DCS run demonstrating the high-resolving power of DCS: As an example, the analysis of a bimodal nanoparticle mixture differing by a few nanometers is shown. The latter contains spherical polystyrene latex particles with number-averaged diameters of 142 nm and 108 nm. It should be noted that the first particle population is identical to the elementary units of the clusters studied in this work, whereas the smaller particles were obtained by increasing the emulsifier concentration during particle synthesis. (**c**) For spherical particles, a particle size distribution is obtained after (i) conversion of the light extinction into particle concentrations using Mie theory, and (ii) recalculation of sedimentation times according to Equation (4).

Differential centrifugal sedimentation (DCS), which is also known as disk centrifuge photosedimentometry (DCP) [13] or as analytical disk centrifugation [14], enables direct measurements of differential size distributions [12]. The method combines centrifugal separation of different nanoparticle populations with continuous light extinction measurement at a fixed position. The centrifugal separation is carried out in a rotating disk (Figure 1a). The latter contains a density gradient, whose densities increase in radial direction. The gradient counteracts a higher apparent density in the sample than in layers underneath and prevents the sample from behaving as a fluid of higher density [15]. This is necessary to make the particles sediment as individual species at rates specified by their sedimentation coefficients. A detector beam is passing the density gradient at a fixed position in the vicinity of the heaviest end of the gradient. As a differential technique, only particles of a specific sedimentation coefficient reach the detector beam and are quantified by the reduction of the detector beam resulting from light scattered by particles. The sedimentation rate of a given particle population depends on its size, buoyant density, and, to a lesser extent, also on its shape [16]. It is for this reason that DCS has been widely used as a highly versatile method for particle sizing in colloid science (Figure 1c). Particle size distributions can be precisely measured down to a few nanometers [12]. This allows for sizing of synthetic and biological particles as diverse as polymer latexes [17], silica particles [13,18], gold nanospheres [18] and nanorods [19], carbon nanotubes, [20], amyloid fibrils [21], and influenza viruses [22], amongst many others.

DCS can be viewed as a complementary method to AUC, which, however, permits fast particle sizing, likewise at excellent resolution, but without the need for solving complicated equations [19]. Further refinement of the instrumentation by the manufacturer in recent years, such as optically, virtually fully transparent disks resistant to organic solvents paired with lower wavelengths of the detector beam, has made DCS a routine tool for measuring particle size distributions at high quality. In addition, substantial scientific progress was made, thereby paving the way for new directions. Armes and co-workers pioneered the analysis of various nanocomposite particles, including sterically stabilized nanoparticles [23] and raspberry-type heteroaggregates [14]. Moreover, the potential to explore colloids with surface-bound proteins was demonstrated [24,25]. Such hybrid particles are characterized by size distributions, which are superimposed by density distributions [14]. Knowledge of the particle size obtained from complementary techniques enables the determination of effective particle densities by DCS [17]. The latter are defined as the difference between the actual particle density and the density of the dispersing medium. DCS measurements thus carried out in density gradients of different composition permit simultaneous determination of particle size and density [13,22]. In the same way, combination of centrifugal sedimentation and flotation triggered by the densities of different dispersing fluids can be used for independent measurements of particle size and density [17].

In this article, we will demonstrate that DCS is a straightforward method to directly measure distributions of sedimentation coefficients in multimodal mixtures of nanoparticles. Colloidal clusters varying in their number of spherical constituent particles and, closely related to this, their geometries will serve as a model system. Such supraparticles are often referred to as “colloidal molecules” as their configurations resemble those of true molecules [26,27,28]. They have well-defined shapes, which are defined by packing criteria for uniform spheres, and can thus be regarded as prototypical for the organization of matter into nanocomposites [27]. The exemplary case study can thus be easily transferred to many other practical problems, including microscopic mechanisms underlying aggregation in aqueous nanoparticle dispersions. In this regard, DCS permits proper discrimination among species differing in their number of constituent particles and provides quick and precise access to sedimentation coefficients. The latter ones can be used to optimize centrifugal routines, enabling effective separation of nanoparticle mixtures.

## 2. Materials and Methods

### 2.1. Chemicals

Ultra-pure D(+)-sucrose (≥99.9%, Proteomics Grade DNAse-, RNAse-free, VWR International GmbH, Darmstadt, Germany) and deionized water (resistivity > 18 MΩ cm) obtained from a reverse osmosis water purification system (Millipore Direct 8, Merck Chemicals GmbH, Darmstadt, Germany) were used for making aqueous density gradients. *n*-Dodecane (≥99%) was purchased from Sigma-Aldrich Chemie GmbH, Steinheim, Germany and used as received. A commercial polystyrene latex standard with a particle diameter of 251 nm (HS0025-20, BS-Partikel GmbH, Mainz, Germany) was used for calibration during DCS experiments.

### 2.2. Colloidal Clusters

The particle clusters studied in this work were prepared by assembly of narrowly dispersed cross-linked polystyrene latex particles while confined at the surface of evaporating emulsion droplets [29]. The polystyrene particles bearing a nanometer-thin hydrophilic surface were synthesized by emulsion polymerization of styrene, divinyl benzene, and *N*-isopropylacrylamide in the presence of sodium dodecyl sulfate as the emulsifier and potassium persulfate as the initiator. A detailed description of the synthesis of the latex particles can be found in [16]. Their average diameter of 144 nm (as determined by transmission electron microscopy) and very low polydispersity index of 1.001 (given by the weight-average diameter divided by the number-average diameter) makes the spherical polymer particles perfectly suited to assembly into well-defined clusters with dimensions in the colloidal regime. The assembly into clusters was accomplished along the lines specified in [16]. The nature of the assembly hinges on trapping a limited number of particles at the surfaces of toluene droplets and packing them into clusters by strong capillary forces that occur during evaporation of the droplet phase [30]. The binding of the particles to the droplets is driven by minimization of surface energy and represents a variation of the Pickering effect with strongly swollen particles at the oil–water interface [31]. The random distribution of the polymer particles across the droplets results in a mixture of clusters differing in the number of constituents, ranging mainly from 2 to 12. In addition, the aqueous suspension of colloidal clusters also contains a fraction of single particles, resulting from droplets bearing just one particle at the surface.

### 2.3. Differential Centrifugal Sedimentation 

Sedimentation coefficient distributions were recorded on an analytical disk centrifuge (DC24000 UHR, CPS Instruments, Inc., Prairieville, LA, USA) [12]. A density gradient was built in situ from 8.0% to 2.0% (m/m) aqueous sucrose solutions by filling the hollow disk rotating at 24,000 rpm. The gradient was covered with a thin layer of *n*-dodecane, thus minimizing evaporation of water and extending the lifetime of the gradient. The step gradient built from nine sucrose solutions (1.6 mL each) was allowed to equilibrate within 30 min, yielding a continuous gradient, which is virtually linear in volume [32].

Diluted sample suspensions (0.02% (m/m)) were sonicated to eliminate any temporary agglomerates and injected into the spinning disk (100 μL in total). The sedimentation time scale of the DCS runs is calibrated with polystyrene nanospheres as a size standard. It must be noted that this calibration is a necessary step in the software of the device before the sample measurement can be performed. However, this calibration had no influence on the actual sedimentation coefficient profiles studied in this work as the latter ones are derived from the raw data (light attenuation at *λ* = 405 nm vs. sedimentation time, cf. Figure 1b) and by using the single particles left in the cluster mixture as an internal reference to determine the constant *k* in Equation (1). By going through this procedure, the sedimentation time scale is finally defined by a component facing the same conditions as the species being investigated in the same DCS run.

### 2.4. Nanoparticle Separation

Preparative separation of the clusters into fractions with the same number of constituent particles was accomplished by rate-zonal density gradient centrifugation [33]. The fundamental procedure corresponds to a large extent the one reported earlier in ref. [16]. However, the focus of the current work was on aligning optimized DCS routines with preparative fractionation as tightly as possible. For this reason, several adjustments became necessary. Sucrose density gradients (36 mL), linear in volume ranging from 2% (m/m) to 8% (m/m), were prepared using a simple gradient maker, originally used for biological separations [34]. The latter is based on two chambers filled with the heaviest and lightest part of the gradient to be built. Gradual mixing of the two sucrose solutions is achieved by a rotating stir bar located in the lighter solution. Gravity results in an outward flow, which is assisted by a peristaltic pump (Masterflex L/S^®^ Digital Miniflex^®^ Pump, Cole-Parmer GmbH, Wertheim, Germany), enabling flowrate (0.1 mL s^−1^)-controlled gradient formation. The latter was built inside a centrifuge tube (Ultra-Clear Centrifuge Tubes, Beckman Instruments, Inc., Fullerton, CA, USA) from below using a drain tube. To facilitate the evaluation of the preparative separation, the centrifuge tubes were equipped with a scale, thus allowing precise allocation of zones of banded particle populations.

Next, 2 mL of cluster suspension (0.36% (m/m)) was carefully placed on top of the density gradient. Centrifugation was performed on an Optima XPN-90 Ultracentrifuge equipped with an SW 32 Ti swinging-bucket rotor (Beckman Coulter GmbH, Krefeld, Germany). Run conditions were 15 min at 24,000 rpm (relative centrifugal forces: RCF_av._ = 70,963, RCF_max._ = 98,703) and 25 °C.

The clusters separated into discrete zones were extracted from the top of the gradient using a self-built fraction recovery system. The tip of a drain tube was directly positioned into the center of the zone of banded particles to be collected. A slight negative pressure was used for gentle extraction. The cluster fractions were dialyzed exhaustively to remove sucrose prior to sample analysis (dialysis membrane: Spectra/Por^®^ 7, MWCO 50,000 kD).

### 2.5. Further Methods

Transmission electron microscopy (TEM) on a Libra 120 microscope (Carl Zeiss AG, Oberkochen, Germany) at an acceleration voltage of 120 kV was used to determine the average size (144 nm) and polydispersity (1.001) of the cluster constituents based on 1000 individual particle counts. The hydrodynamic diameter of the latter is 145 nm, as demonstrated by dynamic light scattering measurements at 25 °C on an ALV/CGS-3 Compact Goniometer System (ALV-Laser Vertriebsgesellschaft m-b.H, Langen Germany). The particle density was derived by measuring the densities of a concentration series using a DMA 5000 M density meter (Anton Paar GmbH, Graz, Austria). Extrapolation of the reciprocal values of the measured densities (= specific volumes) to a solid content of 100% (m/m) yielded a particle density of 1.057 g cm^−3^ at 25 °C. It should be noted that the particle density measured by this method corresponds to the density of the bulk material [35]. However, several studies demonstrated that polystyrene latex particles have buoyant densities, which are identical within the limits of experimental errors to the density specified for the bulk material [36,37]. Fractions of colloidal clusters isolated after centrifugal separation in a density gradient were investigated by field emission scanning electron microscopy (FESEM) using a CrossBeam 1540 XB microscope (Carl Zeiss AG, Oberkochen, Germany) operating at 3 kV.

## 3. Results and Discussion

### 3.1. Analysis of Sedimentation Coefficient Distributions

Figure 1a shows schematically the basic principle of DCS. A mixture of different nanoparticle populations is physically separated into discrete zones of particles that migrate at velocities *u* characteristic to their size, shape, and density. This is achieved within a disk rotating at a constant angular velocity *ω*. Throughout the experiment, two quantities are continuously recorded. One of these is the time *t* it takes distinct particles to travel up a fluid density gradient from a starting position *R*_0_ until they pass a detector position *R*_D._ Arrival of the particles is monitored by the extinction of light at a wavelength of 405 nm. For non-absorbing materials such as polymer particles, the attenuation of a laser beam resulting from light scattered by particles arriving at specified sedimentation times is monitored. 

The sedimentation coefficient *s* of a distinct particle population *i* is the quotient of the sedimentation velocity (*u* = d*R*/d*t*) and the product *ω*^2^*R*, where *R* is the actual radial position with respect to the axis of rotation. *s* is inversely related to the sedimentation time *t_i_* measured in a DCS experiment [19]:

(1) $$s_{i}=\frac{u}{\omega^{2}R}=\frac{\ln\left(\frac{R_{D}}{R_{0}}\right)}{\omega^{2}t_{i}}=\frac{k}{t_{i}},$$

Run-specific parameters such as *R*_0_, *R_D_*, and *ω* can be summarized to one instrumental measurement constant *k*. It is recommended to determine this constant by using a particle standard of known sedimentation coefficient. This has turned out to be beneficial if compared with making measurements by quantifying *R*_0_, *R_D_*, and *ω* individually [8]. 

Polymer particles with spherical shapes are particularly suitable as particle standards because their sedimentation is governed by Stokes’ law [14]. A narrow size distribution will facilitate the calibration process as the local maximum of the distribution, which should correspond to the Stokes diameter of the non-agglomerated particles, can be readily determined. The current work studies mixtures of supracolloidal assemblies built from spherical constituent particles. Consequently, the latter have the same density as clusters based on them. Moreover, these particles are virtually uniform, which is reflected by their low polydispersity index of 1.001. These two conditions make the polymer nanospheres ideal reference particles to precisely determine the constant *k* in Equation (1). To achieve this, the sedimentation coefficient of the particles must be calculated first. 

The sedimentation coefficient of a particle *i* is defined as the ratio of the effective particle mass *m_eff_* and the friction coefficient *f*:

(2) $$s_{i}=\frac{m_{eff}}{f}=\frac{m_{p}-m_{f}}{f},$$

where the effective mass results from the difference in the actual particle mass *m_p_* and the mass of the fluid *m_f_* that is displaced by the particle. For spherical particles, the friction coefficient is given by Stokes’ law and reads as follows:

(3) $$f_{s}=3\pi \eta d_{h},$$

where *η* is the viscosity of the fluid and *d_h_* denotes the hydrodynamic diameter of the particles. The effective mass can be expressed by the corresponding volumes of a sphere with diameter *d_h_* and the densities of the particle *ρ_p_* = 1.057 g cm^−1^ and the density of the displaced fluid *ρ_f_* (here: 0.997 g cm^−3^) [21]:

(4) $$s_{i}=\frac{\left(\rho_{p}-\rho_{f}\right)d_{h}^{2}}{18\eta }.$$

The number-average diameter of the non-agglomerated particles was 142 nm, which was determined by DCS measurement against the commercial polystyrene standard latex. This value is close to the TEM diameter (144 nm) and the hydrodynamic diameter measured by DLS (145 nm).

Due to the small differences related to the low polydispersity of the particles (right peak in Figure 1c represents the size distribution), the values of any of three methods can be used as a measure for the hydrodynamic particle size. The obvious approach would be to use the DCS value of the non-agglomerated species as an internal reference to calculate the sedimentation coefficients of other species present in the mixture (see below).

Alternatively, one might prefer to calculate the masses in Equation (2) from the TEM radius characteristic for dry particles to account for the fact that the hydrodynamic effective surface layer hardly contributes to the particle mass [16]. The friction coefficient given in Equation (3) can be calculated from the hydrodynamic diameter as measured by DCS or DLS. In this study, the DLS value was used to allow for a direct comparison with sedimentation coefficients predicted earlier by hydrodynamic bead-shell modeling. In that study, model building was based on the DLS diameter of the cluster constituents [16].

An important point to note is that by using the non-agglomerated particles as an internal calibration standard, it is guaranteed that any particles present in the same DCS run, whether those to be studied or those acting as a reference, face the same conditions during their migration through the density gradient. This compensation captured in the constant *k* goes beyond the parameters specified in Equation (1). It also considers variations in the density and viscosity of the dispersion medium during sedimentation. This is particularly important when studying sedimentation in a density gradient. In other words, a compensation for fluid densities and viscosities that differ from the actual conditions during the experiment is achieved by using an internal reference. The sedimentation characteristics within the gradient can thus be easily transferred to the sedimentation in other dispersion media. In the following, calculation of the sedimentation coefficient of the reference particles is based on the sedimentation in pure water at 25 °C (*ρ_f_* = 0.997 g cm^−3^; *η* = 0.891 g m^−1^ s^−1^). The latter is done not only for the sake of simplicity, but also to allow for direct comparison to predicted values from hydrodynamic modeling that have been reported previously [16]. Consequently, a sedimentation coefficient of *s_N_*_=1_ = 770 Sv is calculated from Equation (4). The sedimentation time of the nanospheres assigned from the DCS analysis of cluster mixtures is *t_N_*_=1_ = 401 s, which yields a *k*-value of 3.0877 × 10^−8^ according to Equation (1). This value is then used to calculate the sedimentation coefficients of any non-spherical species present in the nanoparticle mixture from their respective sedimentation times.

### 3.2. Particle Clusters as Model Systems for Nanoparticle Mixtures

Thanks to the seminal work by Pine and co-workers [30], it is known that evaporating emulsion droplets are suitable physical templates to assist the assembly of colloidal particles into an ensemble of stable clusters varying in their number of constituents. A mixture of various species is obtained, resulting from a random distribution of the elementary particles on the droplets [31]. The gradual increase in cluster mass follows directly from the number of constituent particles *N*. A linear correlation does not apply to the increase in surface area and, related to this, friction at the cluster surface. The growth in surface area is largest when going from single particles to dimers, followed by increasingly smaller growth rates with rising *N*. The latter is a direct consequence of the packing into dense cluster configurations. It is obvious that the sedimentation coefficients of the clusters gradually increase with rising *N* in accordance with Equation (2). Moreover, based on the above, the increments for the rising sedimentation coefficients are becoming increasingly smaller with rising *N*. This fact underscores the suitability of particle clusters as model systems to explore the limits of sorting nanoparticle mixtures by centrifugal sedimentation.

**Figure 2:**
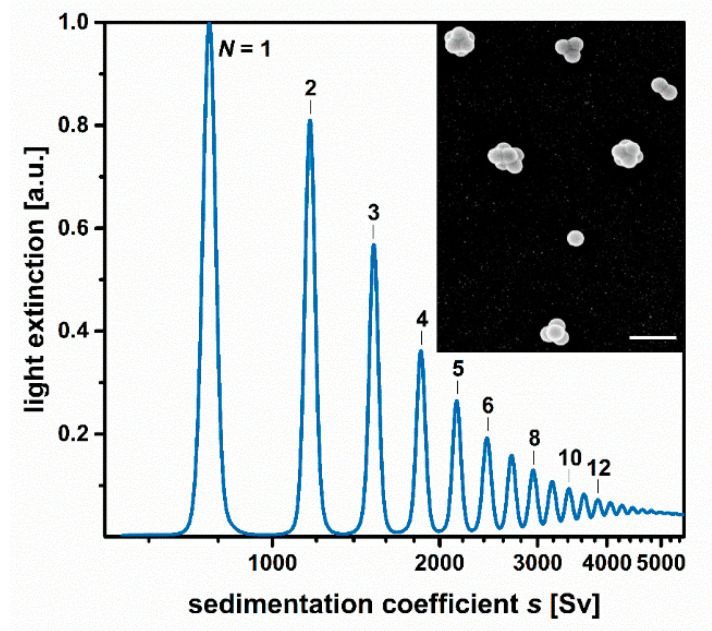
Sedimentation coefficient distribution of a mixture of colloidal clusters as measured by DCS. Cluster species of more than 12 constituent particles are resolved separately as narrow bands. DCS analysis thus enables a clear classification of nanoparticles according to their sedimentation characteristics. The inset shows an FESEM micrograph of the cluster mixture studied by DCS. The scale bar represents 400 nm.

**Figure 3:**
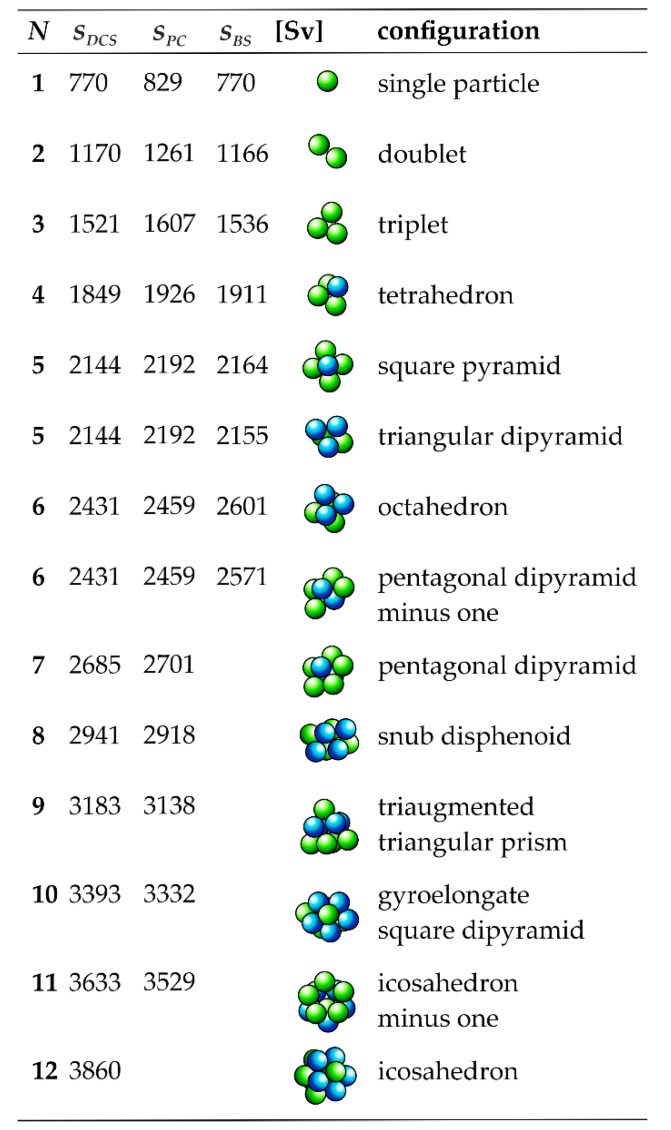
Sedimentation coefficients of the cluster species varying in the number of constituent spheres *N* and, consequently, their configurations. Analytical values *s_DCS_* obtained from DCS are compared with sedimentation coefficients S*_PC_* estimated from preparative separations by rate-zonal density gradient centrifugation. In addition, sedimentation coefficients *S_BS_* predicted from hydrodynamic bead-shell modeling, as taken from [16], are given for clusters with *N* ≤ 6. Model building considers the exact geometries of the colloidal clusters. Notwithstanding the shortcomings in deriving sedimentation coefficients from preparative centrifugations, the quantities thus obtained differ by less than 8% from the analytical values and the theoretical predictions.

The following studies are based on a mixture of particle clusters built from 144 nm-sized polymer nanospheres (Inset of Figure 2). Most of the clusters consist of up to 12 constituent particles and thus have spatial dimensions within the colloidal domain. Hence, Brownian motion will prevail over sedimentation unless the assemblies are exposed to a centrifugal field [38]. The morphology of the colloidal clusters in this work and of related supraparticles was discussed in earlier works [16,28,30,31,39,40]. It should be noted that clusters of more than four constituents may occur in different configurations. For example, clusters of five and six particles have two different configurations. Figure 3 shows common configurations that are observed experimentally and predicted by computer simulations. The cluster mixture contains both isotropic and anisotropic species. Single particles, tetrahedral, octahedral, and icosahedral clusters have isotropic geometries, whereas all other species exhibit anisotropic shapes. The further studies will thus deliberately dispense on any evaluations based on assuming a spherical particle geometry. The basic methodology is thus broadly applicable to particles of arbitrary shapes that do not have to obey Stokes’ law.

### 3.3. DCS Analysis of Sedimentation Coefficient Distributions

DCS is now applied as an analytical tool to explore the distribution of sedimentation coefficients of the mixture of colloidal clusters. The separation of the particles within the disk centrifuge follows the basic principles of rate-zonal density gradient centrifugation [15]. The proper design of the density gradient is key for accurate results. The following criteria must be considered when preparing the density gradient:

1. The density of the particles must exceed the highest density within the gradient.
2. The density of the sample suspension (particles + dispersion medium; here: 0.997 g cm^−3^) has to be lower than the lowest density within the gradient.

Compliance with the two criteria makes the particles settle individually at rates specified by their sedimentation coefficients. In the present case, clusters of a single set of constituent particles were explored at 25 °C. Hence, the various species within the cluster mixture have the same density, which is 1.057 g cm^−3^. Their separation during DCS analysis was performed in an aqueous sucrose gradient ranging from 2% (m/m) to 8% (m/m). The minimum density within the gradient equals 1.0052 g cm^−3^ and is thus, in accordance with criterion 2, higher than the density of the highly diluted sample suspension (0.02% (m/m), *ρ* = 0.997 g cm^−3^). This prevents streaming, i.e., a downstream of particle-laden fluid following the centrifugal field [15]. In addition, the maximum density within the gradient (1.0285 g cm^−3^) was kept lower than the actual particle density. This ensures that the particles can travel though the complete gradient fluid and will thus reach the detector position at a characteristic sedimentation time. The gradient design suitable for polystyrene latex particles can be easily adapted for other types of nanoparticles, simply by following the two criteria. The densities of aqueous sucrose solutions as functions of weight fraction and temperature can be found in the literature [41]. Figure S5 displays densities in the concentration range up to 10% (m/m) sucrose.

The particle clusters do not absorb visible light, but light is scattered by the clusters, as for any other colloidal objects. The colloidal clusters can be thus detected by attenuation of a laser beam. The latter had a wavelength of 405 nm, which provides high sensitivity because violet light is subject to the strongest scattering within the visible region. For this reason, even marginal quantities of particles or particle agglomerates can still be detected.

During the DCS run, the light attenuation of the detector beam is continuously recorded as the function of the sedimentation time. Routines in the device software allow for conversion of the raw data into a particle size distribution. These routines are based on the Mie theory to derive the particle concentration from the measured light extinction [42]. Moreover, the device software assigns a distinct particle diameter to any given sedimentation time. However, this correlation is only correct if the drag force acting on the particles during sedimentation follows Stokes’ law. The latter applies only to spherical particles and is thus not appropriate for accurately exploring particles with complex shapes. The deviation between the friction coefficients of an anisotropic particle and a spherical particle of the same mass can be at least partially compensated by the introduction of a non-sphericity factor [12]. However, this can only work if all the particles exhibit the same aspect ratio, which is rarely the case for mixtures of anisotropic particles. Nanoparticle aggregates such as the colloidal clusters in this study have various geometries (Figure 3), so that the assignment of a single non-sphericity factor that works for all species is not possible.

It is exactly for this reason that the present evaluation explicitly avoids any assumptions of a spherical shape and is thus applicable to particles of arbitrary shapes. The only exception is made when calculating the sedimentation coefficient of the spherical particles used as an internal reference. In this specific case, making use of Equation (3) is justified. Basically, it is also possible to replace the spherical particles by any other type of particles given that their sedimentation coefficient is known. Precise knowledge of the sedimentation coefficient of at least one set of reference particles is required to determine the instrumental measurement constant *k* in Equation (1). These reference particles can be either measured independently of the sample particles, or, as in the present case, constitute a distinct particle population within the nanoparticle mixture. The latter procedure is advantageous as different particle populations are compared, which are subject to the same experimental conditions. In case of nanoparticle aggregates, it makes sense to use the elementary particles as an internal reference. The *k*-value calculated from the sedimentation time of the reference particles and their sedimentation coefficient can thus be transferred to any other particle population within the same DCS run. Sedimentation coefficients of the different particle populations are calculated from the respective sedimentation times according to Equation (1). 

Together with the profile of light extinction measured during DCS, a distribution of the sedimentation coefficients of the nanoparticle mixture is obtained. This distribution is weighted by the attenuation of light caused by the particle arriving at the detector position and thus differs from the weight distribution *c*(*s*) obtained from AUC [43]. In principle, conversion of the two contributions is possible if the scattering cross-sections of the various particle populations are known. According to the authors’ opinion, this issue should be treated in a purist way to allow for a broad application of the methodology.

Figure 2 shows the light extinction-weighted sedimentation coefficient distribution of a mixture of colloidal clusters. As described above, the distribution is solely based on the raw data of the DCS run and knowledge of the sedimentation coefficient of the single particles used as an internal reference. The high resolution of the DCS run is immediately obvious. Single particles and cluster species of 2 to 12 constituents are resolved as discrete bands, which allows for a facile assignment of sedimentation coefficients according to the peak maxima (Figure 3). Remarkably, species that deviate by less than 1% in their sedimentation coefficients can still be resolved. A more detailed discussion of the resolution of DCS is found in ref. [12].

**Figure 4:**
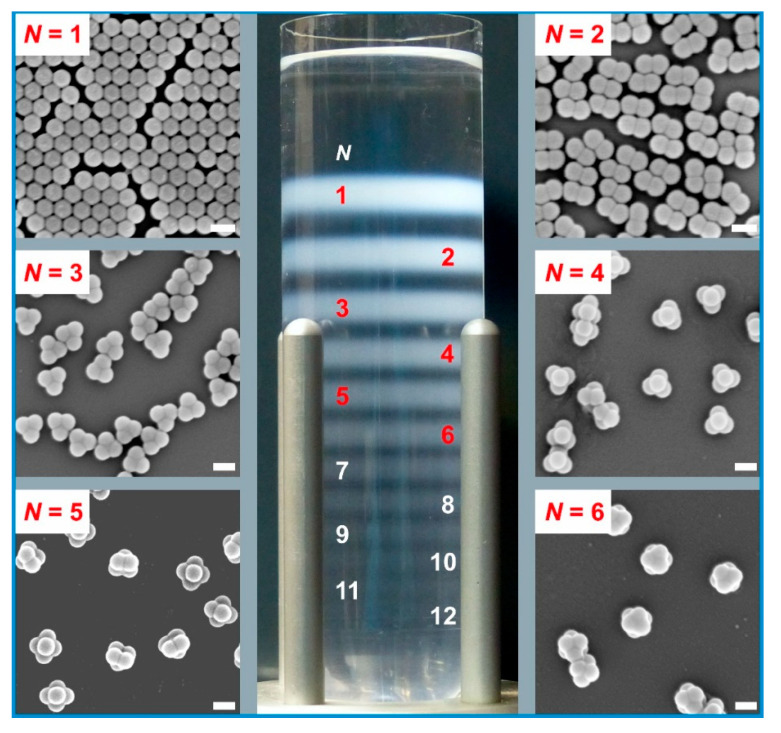
Centrifugal separation of colloidal clusters according to their sedimentation coefficients in a sucrose density gradient ranging from 2% (m/m) to 8% (m/m). The fractionation was carried out in a swing-out rotor at 24,000 rpm. Cluster populations of up to 12 constituent particles were isolated as individual zones that could be harvested by using a self-built fraction recovery unit. FESEM micrographs of fractions of particle monomers (*N* = 1), dimers (*N* = 2), trimers (*N* = 3), tetramers (*N* = 4), pentamers (*N* = 5), and hexamers (*N* = 6) are grouped around the centrifuge tube hosting the gradient and the particle zones. The scale bars represent 200 nm.

Zone widths in a DCS experiment are governed by several factors. Because DCS detects particles arrive at a fixed position over time, the actual zone width of a given particle population is captured as a time interval within which all particles of this population arrive at the detector position. A broad zone is thus reflected by a larger time interval. This becomes apparent when the light extinction is plotted against the sedimentation time (Figure S1). The peak-width at half-height is largest for the non-agglomerated particles (25.2 s) and decrease systematically for the clusters with rising numbers of constituent particles *N* (13.0 s for dimers; 9.2 s for trimers; 7.3 s for tetramers; 6.0 s for pentamers; 5.4 s for hexamers). The polydispersity of the cluster constituents (1.001) and, consequently, of the clusters is rather low and virtually negligible compared to the broadening due to Brownian motion. Band broadening due to diffusion is an important aspect. Translational diffusion coefficients decrease with the number of constituent spheres [16,38] and, consequently, facilitate band narrowing with rising numbers of constituent particles *N* (Figure S1). The density gradient influences the band width as well. A steep gradient profile results in lower sedimentation velocities in the leading edge of the zone, whereas the velocities are higher at the trailing edge. The density gradient thus keeps the zone of a distinct particle population together. The density gradient equally affects all particle populations because the detector position is fixed in DCS measurement. This constitutes a difference from preparative centrifugal separations, where different particle populations are harvested from different sections of the density gradient (Figure 4).

According to Equation [2], there is an inverse relationship between sedimentation time and sedimentation coefficient. This must be considered when comparing actual zone widths during the DCS run with the widths of the individual peaks in the sedimentation coefficient distribution (Figure 2). It follows that the non-agglomerated particles which form the broadest zone (peak-width at half-height: 25.2 s) during the DCS run are presented by the narrowest peak in the sedimentation coefficient distribution (peak-width at half-height: 48 Sv). In comparison, the tetramers form narrower zones (peak-width at half-height: 7.3 s) but are represented by a broader peak in the sedimentation coefficient profile (peak-width at half-height: 81 Sv).

Accurate assessment of the sedimentation coefficients obtained from the DCS strategy can be based on a comparison with values obtained from theoretical modeling. García de la Torre and co-workers have established model building and calculation routines, which can be applied to rigid particles of arbitrary shapes. Recently, we reported on sedimentation coefficients of colloidal clusters calculated along these lines [16]. The clusters studied are identical to those in the present studies but limited to a maximum of six constituent particles. Nonetheless, a direct comparison can be made (Figure 3). The sedimentation coefficients obtained by DCS are in excellent correlation to the values predicted for clusters with the same spatial dimensions and geometries. Deviations are negligible for single particles and even for dimers, which are the geometries with the highest aspect ratio. Although the deviations increase with the number of constituents, they are less than 6% for six-particle clusters with octahedral symmetry. This underscores the suitability of DCS to directly measure sedimentation coefficient distributions of complex nanoparticle mixtures.

### 3.4. Application for Preparative Nanoparticle Separations

The separation quality accomplished during the DCS analysis should now serve as a basis to optimize preparative centrifugal separations. To this end, separations according to the sedimentation coefficients were carried out in a swinging-bucket rotor. The buckets host clear centrifuge tubes with a capacity of 38.5 mL. Compared to a fixed-angle centrifuge rotor, swing-out rotors significantly reduce collisions of the particles with the wall of the tube and help to reduce wall effects on sedimentation (see below). More importantly, the fluid layers and the particle zones follow the centrifugal field throughout the whole run, including acceleration and deceleration intervals, which is the prerequisite for excellent selectivity during fractionation. The orientation of the layers with respect to the axis of rotation is a feature which DCS analysis and separations carried out in a swing-out rotor have in common. In this context, the spinning hollow disk in DCS can be considered as an extension of the centrifuge tube in a swing-out rotor by 360 °C (Figure 1).

Nevertheless, there are two important differences that should be kept in mind. The first one is related to the geometry of the disk, which has some similarities to a zonal rotor used for preparative separations at larger scales [32]. A density gradient linear in volume will be also linear along the sedimentation path if placed in a centrifuge tube. However, if prepared within the rotating hollow disk, a concave profile of the density gradient is obtained across the sedimentation path. The second difference to be considered relates to the substantial difference between an analytical technique and a preparative method. DCS probes the different particle populations after having reached a fixed position. This means that all particles have migrated along the same path, but they have reached their destination at different times according to their sedimentation coefficient. In preparative separations, the principle of operation is reversed. The centrifugal run and thus the sedimentation are terminated for all particle populations at the same time. At precisely this time, they have traveled different distances, which are determined by their sedimentation coefficients. Hence, the two methods offer the opportunity to complement each other if aligned at the same experimental problem.

Parallel to the above-mentioned DCS analysis, sucrose density gradients ranging also from 2% (m/m) to 8% (m/m) were prepared in centrifuge tubes. In line with the DCS analysis, the centrifugation was performed at 24,000 rpm. Centrifugation times were chosen based on the sedimentation coefficients measured by DCS. In doing so, the time required to maximize the sedimentation path of a 12-particle cluster was estimated. This ensured maximum separation of the cluster populations. 

Figure 4 shows the separation achieved in the centrifugal run. A total of twelve discrete zones, each of them corresponding to a particle population settling at the same rate, are observed. Classification of the zones to specific clusters follows the number of constituent particles *N*. This is in full accord with the sequence of sedimentation coefficients obtained from DCS (Figure 3). Verification of zone allocation was achieved by FESEM after extraction of the cluster fractions (Figure 4).

The separation of the cluster mixture into discrete zones within the centrifuge tube paved the way for a quantitative evaluation. There are a number of reports in the literature on the calculation of sedimentation coefficients from separations of biomacromolecules performed in a centrifuge tube [15,44,45]. According to Equation (1), it is possible to calculate sedimentation coefficients from the radial locations of the various zones once the centrifugal run is terminated. To this end, a centrifugal separation of the cluster mixture was carried out in a graduated test tube. The *R_D_* values in Equation (1) are taken from the center of mass of the zones. The initial position *R*_0_ is assumed as the interface between sample zone and density gradient (Figure S3). The effective time of centrifugation was determined along the lines given by Schumaker [15]. To this end, the course of the rpm values during the entire centrifugal run was recorded. Periods of acceleration and deceleration are considered by plotting the angular velocity-squared ω^2^ with the course of the centrifugation time (Figure S2). Integration of *ω*^2^ over the entire time span and division by the maximum value of $\omega_{max}^{2}$ gave an effective centrifugation time of 470.4 s at maximum angular velocity *ω_max_* (here: 2513.4 s^−1^). 

The calculation according to Equation (1) will yield the actual sedimentation coefficients of the different nanoparticle populations within the density gradient. The latter can be considered as dispersion medium with a gradual change not only in density but also in viscosity. In DCS, the impact of the medium is considered by the measurement constant *k*. Hence, sedimentation coefficients determined by DCS reflect experimental conditions identical to those assumed for the particles used as a calibration standard. The data gathered in Figure 3 refer to the sedimentation in pure water at 25 °C. To allow for a comparison with the DCS values, it is necessary to correct the values determined from preparative separations by the mean viscosities and densities, which the particles experience during their sedimentation. This correction can be made by using the following expression [44]:

(5) $$s_{PC}=s_{G}\cdot \frac{\rho_{p}-\rho_{W}}{\rho_{p}-\rho_{G}}\cdot \frac{\eta_{G}}{\eta_{W}},$$

where *s_PC_* and *s_G_* denote the sedimentation coefficients of a given particle population at 25 °C in pure water and in the gradient. *ρ_W_* = 0.997 g cm^−3^ and *η_w_* = 0.891 g m s^−1^ are the density and viscosity of water at 25 °C.

The mean density and viscosity of the gradient, *ρ_G_* and *η_G_*, were determined as follows: first, the sucrose concentrations at the zone centers were calculated based on the linear profile of the gradient (Figure S3). Densities and viscosities of aqueous sucrose solutions given in ref. [41] were plotted against the weight proportion of sucrose and subjected to a polynomial fitting (Figures S4 and S5). The fitting functions were integrated from the minimal concentration of sucrose in the gradient (2% (m/m)) to the concentration of sucrose at the zone center (Figure S3). Division by the difference in the two sucrose concentrations yields the mean density and viscosity during the sedimentation of a given particle population. In the concentration range considered, the densities and viscosities increase virtually on a linear scale (Figures S4 and S5). Hence, the derivation of the quantities *ρ_G_* and *η_G_* could be also simplified by averaging the respective quantities at the beginning and at the end of the sedimentation path.

**Figure 5:**
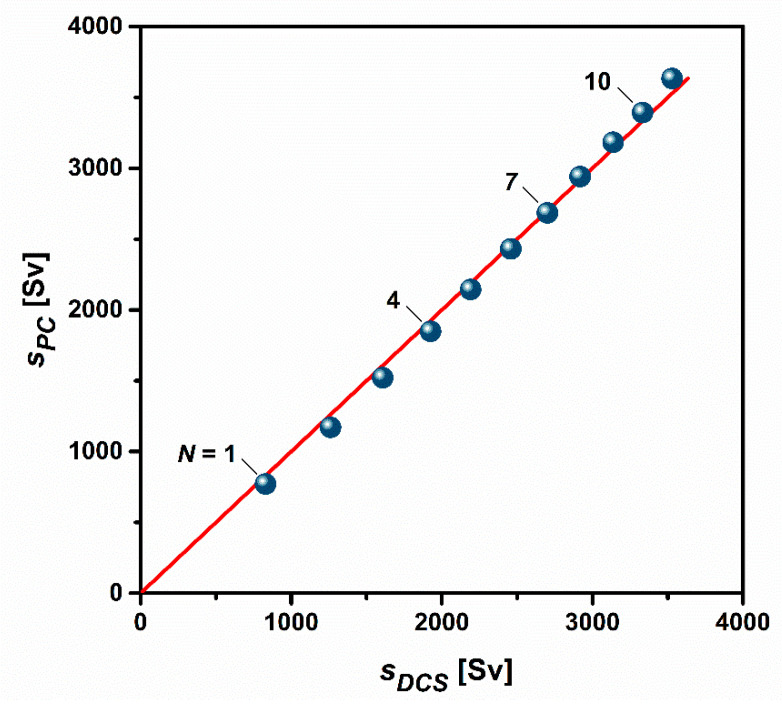
Cross-comparison of sedimentation coefficients shows excellent agreement between the quantities determined by DCS and those estimated from preparative centrifugal separation. The bisecting red line is added for ease of classification and to indicate deviations from full convergence.

Figure 5 shows a comparison of the sedimentation coefficients *s_PC_* calculated from the radial positions of the zone centers within the centrifuge tube with the quantities determined by DCS. Notably, the sedimentation coefficients obtained by the two strategies deviate by less than 8%. This is remarkable, inasmuch as there are a number of uncertainties in the calculation of accurate sedimentation coefficients from the results of rate-zonal separations carried out in centrifuge tubes [46]. The following issues must be considered:

1. uncertainty in the particle density;
2. uncertainties in the profile of the density gradient;
3. uncertainties in the positions of the zones;
4. temperature variations inside the rotor chamber;
5. wall effects.

This study has shown that deviations in comparison to other methods can be kept low. It is briefly outlined how this was achieved despite the uncertainties listed above.

This study is centered on the determination of the sedimentation of solid nanoparticles. In contrast to most biological particles or synthetic micro- or nanogels, solid particles do not vary their density while migrating through a density gradient. Uncertainty in particle density is thus limited to experimental errors in the determination of particle densities. The latter can be precisely measured with an uncertainty of less than 0.005 g cm^−3^.

Considerable uncertainties in the density gradient profile shown in Figure S3 are to be expected in the top layer of the gradient underneath the sample zone. Diffusion of gradient material (here: sucrose) into the sample zone may cause strong deviations from the expected gradient profile [32,46]. However, it turned out that this phenomenon had little effect on the calculated values of the sedimentation coefficients. This was achieved by setting an effective sedimentation time, allowing the zones of migrating particles to reach positions far from the first layers of the gradient (Figure S3).

Uncertainties in radial positions relate to both the starting zone and the final positions of the zones of banded particles. Precise allocation of the beginning of the sedimentation path is challenging inasmuch as the profile of density gradient is not well known at the interface between the sample zone and the top layer of the gradient. It may thus be justified to define the starting position either by the interface itself or as the center of mass within the sample zone. In the present case, the two positions differed by 2.1 mm. This value can be reduced by smaller sample volumes at the expense of the number of particles that can be separated in a single run. In this work, the nanoparticles were dispersed in pure water. For this reason, streaming is to be expected within the sample zone. This is why the diverse nanoparticle populations rapidly accumulate near the interface of sample and gradient during acceleration of the centrifuge. It is exactly for this reason that the radial position of the beginning of the gradient (*R* = 70.14 mm) was chosen as the beginning of the sedimentation path for all nanoparticle populations. This definition was corroborated by the agreement with the values from DCS and hydrodynamic modeling.

The sedimentation coefficients were calculated from the radial positions of the centers of mass of the zones, which are affected by the centrifugal field. As a result, the particle distributions deviate from Gaussian profiles. In the present case, uncertainty of the zone position had little effect because of the low bandwidths resulting from the narrow size distributions of the particle populations. Nonetheless, further refinement could be achieved by taking into account the interplay of sedimentation and diffusion along the lines given by Schumaker [15].

Variation in the temperature inside the rotor chamber may have a marked effect on the sedimentation of the particles. An uncertainty of 1 °C will cause an error of over 2.5% in the determination of sedimentation coefficients [46]. Variations in temperature affect both the density and viscosity of the gradient and can become a major problem when calculating sedimentation coefficients from centrifugal separations. In the DCS measurements shown above, this problem was circumvented by using an internal calibration standard of known sedimentation coefficient. It is also possible to use the same strategy in preparative centrifugal separations. Alternatively, values obtained from preparative separations can be corrected by DCS analysis of at least one component. In the present case, this was not done to identify deviations among the methods.

Collisions of nanoparticles with the walls of cylindrically shaped centrifuge tubes may occur. Particles that hit the wall may stick to it or accumulate near the wall and then settle down as an ensemble at modified sedimentation velocities [15,47]. Only those particles that escape from collision with the side wall, exhibit ideal sedimentation behavior. The latter fraction is larger if the centrifuge tube is placed in a swinging bucket following the direction of the centrifugal field. Further improvement can be achieved by using either radially shaped centrifuge tubes [15] or zonal rotors that avoid wall effects by allowing the sedimentation to proceed within a bowl-shaped chamber [32].

Notwithstanding these uncertainties, the present results have shown that close agreement with sedimentation coefficients, either experimentally measured by DCS or predicted by theoretical modeling, can be achieved. Consequently, the three rather different approaches in determining sedimentation coefficients complement each other very well. Sedimentation coefficient distributions, which are readily accessible by DCS, have proven a valuable tool to optimize the centrifugal separation of nanoparticle mixtures.

## 4. Conclusions

The agglomeration of nanoparticles yields a large variety of supraparticles, which differ in their aggregation numbers, their compositions, their spatial dimensions, and, finally, their shapes. Clusters of a limited number of nanoscale constituents underlie Brownian motion and have well-defined geometries if prepared by a template-based assembling strategy. This makes the latter ideal model systems for testing analytical tools that are suitable for exploring multimodal mixtures of complex nanoparticles. The latter is challenging and time-consuming with common analytical methods. This is because they provide either poor statistics, such as electron microscopy, or require separation into monomodal fractions prior to their use. The strength of DCS to investigate multimodal mixtures lies in the fact that DCS performs the analytics online, while the nanoparticle mixture is being separated into its individual components. The application of DCS was demonstrated by the analysis of mixtures of colloidal clusters. Nanoparticle clusters of more than 12 constituent particles could be resolved as discrete bands. This, in turn, allowed for an immediate allocation of the observed bands to distinct species. Without any sophisticated mathematical deconvolution, DCS gave access to accurate sedimentation coefficient distributions, simply by using the elementary units of the assemblies as an internal reference. In this way, DCS facilitates an easy-to-use and efficient analysis of colloids with anisotropic shapes, which do not obey Stokes’ law. This was verified by comparison with theoretical predictions of sedimentation coefficients for various species of the multimodal mixture by hydrodynamic bead-shell modeling, which considers the exact geometry of the species. 

The sedimentation coefficient distributions determined by DCS can be used to optimize routines for preparative centrifugal separations. This study has shown the separation of a total of 11 different populations of colloidal molecules into defined zones within a sucrose density gradient. In addition, the estimation of sedimentation coefficients from the locations of the zones was demonstrated. In this context, the present work could extend an approach, originally established in biological separations, to synthetic nanoparticles. 

The feasibility of DCS analysis and its practical application to optimize centrifugal separations suggests the broad applicability of DCS to other nanoparticle systems, including irregularly shaped colloids or mixtures of compositionally heterogeneous nanoparticles.

## Data Availability

The datasets generated during and/or analyzed during the current study are available from the corresponding author on reasonable request.

## Supplementary Materials

The following are available online at https://www.mdpi.com/article/10.3390/nano11041027/s1, Figure S1: DCS raw data: light extinction recorded as the function of the sedimentation time, Figure S2: Centrifugal run log to estimate the effective time of centrifugation, Figure S3: Profile of the sucrose density gradient built in the centrifuge tube, Figure S4: Viscosities of aqueous sucrose solutions as functions of temperature and concentration; Figure S5: Densities of aqueous sucrose solutions as functions of temperature and concentration.
