# Supplementary material for: Direct Measurement of Sedimentation Coefficient Distributions in Multimodal Nanoparticle Mixtures
Source: Nanomaterials (Basel). 2021 Apr 17;11(4):1027. doi: 10.3390/nano11041027 (PMC8072784; doi:10.3390/nano11041027)
Supplement: Supplementary file 1 [file nanomaterials-11-01027-s001.zip › nanomaterials-1167771-SI.pdf]

Supplementary Materials

Direct Measurement of Sedimentation Coefficient Distributions in Multimodal Nanoparticle Mixtures

Claudia Simone Plüisch, Rouven Stuckert and Alexander Wittemann *

Supplementary Figures

The following figures illustrate the derivation of the parameters required to calculate sedimentation coefficients from preparative centrifugal separations. See text of the main article for further explanations.

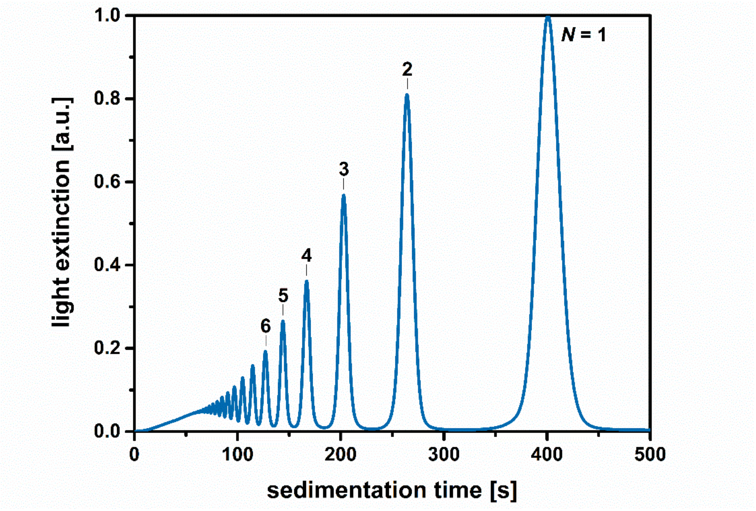

Figure S1. A mixture of colloidal clusters as measured by differential centrifugal sedimentation (DCS). The light extinction is recorded continuously as the function of the sedimentation time. *N* denotes the number of constituent particles of the clusters. The widths of the peaks are related to the widths of the zones of distinct particle populations arriving at a fixed detector position.

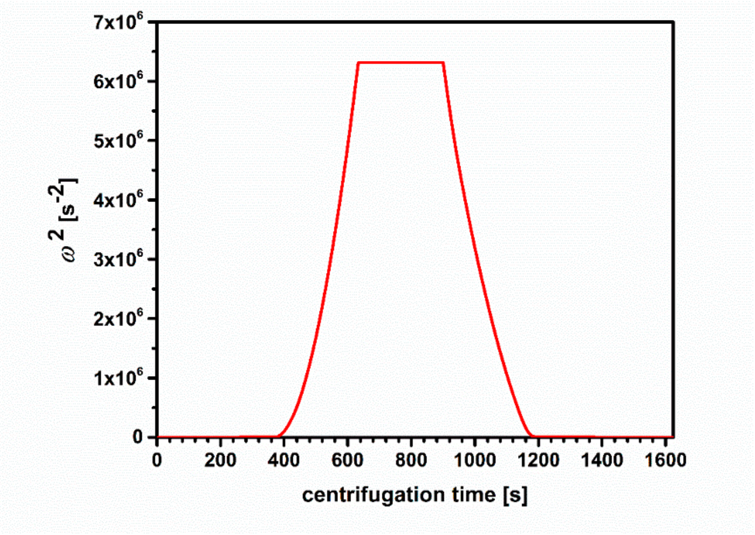

Figure S2. Estimation of the effective time of centrifugation along the lines given in [1]. Angular velocities-squared ω^2 are plotted against centrifugation time. Integration over time and division by maximum angular velocity-squared gives the equivalent time to centrifugation at maximum speed, which is regarded as the effective time of centrifugation.

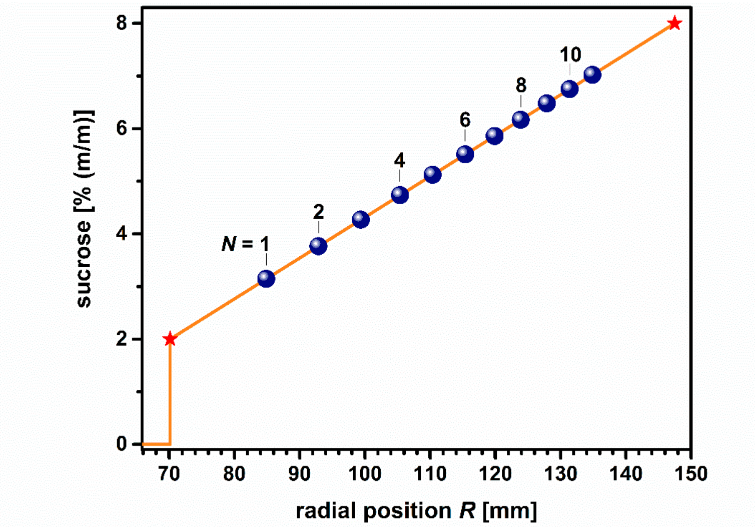

Figure S3. The graph shows the separation that is achieved in sorting colloidal clusters of up to eleven constituent particles via density gradient ultracentrifugation. The asterisks mark the onset (2% (m/m) sucrose) and the end (8% (m/m) sucrose) of the linear sucrose gradient. The cluster suspension was put on top of the gradient and centrifuged for 15 min at 24,000 rpm at 25 °C. The blue spheres indicate the radial positions of the individual bands within the gradient after centrifugation (*N* = 1:3.14% (m/m), 84.89 mm; *N* = 2:3.76% (m/m), 92.89 mm; *N* = 3:4.27% (m/m), 99.39 mm; *N* = 4:4.73% (m/m), 105.39 mm; *N* = 5:5.12% (m/m), 110.39 mm; *N* = 6:5.51% (m/m), 115.39 mm; *N* = 7:5.86% (m/m), 119.89; *N* = 8:6.17% (m/m), 123.89 mm; *N* = 9:6.48% (m/m), 127.89 mm; *N* = 10:6.75% (m/m), 131.39 mm; *N* = 11:7.02% (m/m), 134.89 mm).

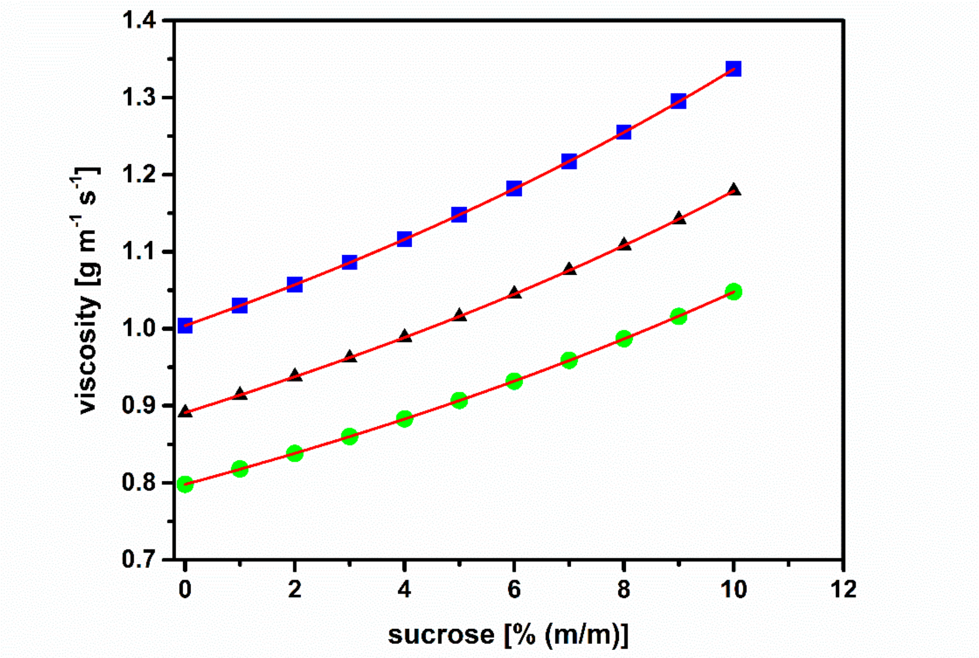

Figure S4. Viscosities of sucrose aqueous solutions at 20 °C (blue squares, fit curve: y = 1.00399 + 0.02533x + 5.89161 × $10^{-4}x^2$ + 2.07848 × $10^{-5}x^3$), 25 °C (black triangles, fit curve: y = 0.89105 + 0.02247x + 3.85781 × $10^{-4}x^2$ + 2.44755 × $10^{-5}x^3$) and 30 °C (green spheres, fit curve: y = 0.79803 + 0.01925x + 4.34149 × $10^{-4}x^2$ + 1.37918 × $10^{-5}x^3$). The viscosities as the functions of the concentration and the temperature were taken from [2].

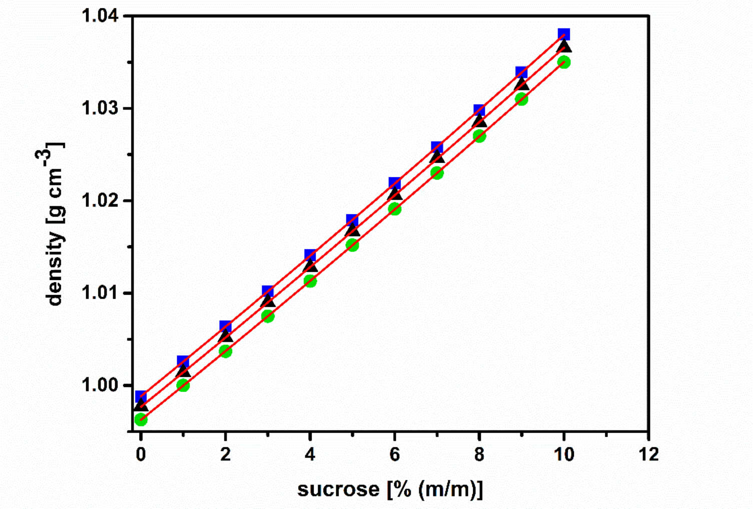

Figure S5. Densities of sucrose aqueous solution at 20 °C (blue squares, fit curve: $y = 0.99884 + 0.00373x + 1.84149 \times 10^{-5}x^2$), 25 °C (black triangles, fit curve: $y = 0.99769 + 0.00371x + 1.74825 \times 10^{-5}x^2$) and 30 °C (green spheres, fit curve: $y = 0.99628 + 0.00369x + 1.88811 \times 10^{-5}x^2$). The densities as the functions of the concentration and the temperature were taken from [2].

References

1. Schumaker, V.N. Zone Centrifugation. In *Advances in Biological and Medical Physics*, Lawrence, J.H., Gofman, J.W., Eds.; Academic Press: New York, NY, USA, 1967; Volume 11, pp. 245–339, doi:10.1016/B978-1-4832-3107-5.50011-X.
2. Price, C.A. Appendix C—Properties of Gradient Materials. In *Centrifugation in Density Gradients*, Price, C.A., Eds.; Academic Press: London, UK, 1982; pp. 327–389, doi:10.1016/B978-0-12-564580-5.50017-9.
